# Transformational leadership, transnational culture and political competence in globalizing health care services: a case study of Jordan's King Hussein Cancer Center

**DOI:** 10.1186/1744-8603-3-11

**Published:** 2007-11-16

**Authors:** Jeffrey L Moe, Gregory Pappas, Andrew Murray

**Affiliations:** 1 Fuqua School of Business, Duke University, Box 90120, Durham, NC, 27708-0120, USA; 2 Department of Community Health Sciences, Aga Khan University, 3700 Stadium Road, Karachi, Pakistan; 3 Discovery Care, Johannesburg, South Africa

## Abstract

**Background:**

Following the demise of Jordan's King Hussein bin Talal to cancer in 1999, the country's Al-Amal Center was transformed from a poorly perceived and ineffectual cancer care institution into a Western-style comprehensive cancer center. Renamed King Hussein Cancer Center (KHCC), it achieved improved levels of quality, expanded cancer care services and achieved Joint Commission International accreditation under new leadership over a three-year period (2002–2005).

**Methods:**

An exploratory case research method was used to explain the rapid change to international standards. Sources including personal interviews, document review and on-site observations were combined to conduct a robust examination of KHCC's rapid changes.

**Results:**

The changes which occurred at the KHCC during its formation and leading up to its Joint Commission International (JCI) accreditation can be understood within the conceptual frame of the transformational leadership model. Interviewees and other sources for the case study suggest the use of inspirational motivation, idealized influence, individualized consideration and intellectual stimulation, four factors in the transformational leadership model, had significant impact upon the attitudes and motivation of staff within KHCC. Changes in the institution were achieved through increased motivation and positive attitudes toward the use of JCI continuous improvement processes as well as increased professional training. The case study suggests the role of culture and political sensitivity needs re-definition and expansion within the transformational leadership model to adequately explain leadership in the context of globalizing health care services, specifically when governments are involved in the change initiative.

**Conclusion:**

The KHCC case underscores the utility of the transformational leadership model in an international health care context. To understand leadership in globalizing health care services, KHCC suggests culture is broader than organizational or societal culture to include an informal global network of medical professionals and Western technologies which facilitate global interaction. Additionally, political competencies among leaders may be particularly relevant in globalizing health care services where the goal is achieving international standards of care. Western communication technologies facilitate cross-border interaction, but social and political capital possessed by the leaders may be necessary for transactions across national borders to occur thus gaining access to specialized information and global thought leaders in a medical sub-specialty such as oncology.

## Background

Globalization, some argue "internationalization," [1] is occurring across many industries, and with increasing frequency and magnitude in health care services [2,3]. New technologies have made cross-border economic transactions, communication and data exchange less expensive, more broadly available and more applicable to health care requirements. Health care services have become "tradable" through international commercial arrangements and sanctioned by global trade policies (e.g. General Agreement on Trade Services through the World Trade Organization). Yet the full extent of globalizing health care services (GHCS) includes government to government activity and increasingly non-governmental organizations. Multi-national health-related initiatives, both public and private, are resourced through direct supply of health care technology, staff, goods and services [4,5] as well as funding. The exchange or purchase of health care services from wealthy to developing and poor countries is also increasing [6,7]. Motivated by economic gain, foreign policy interests or simple human compassion, an unprecedented expansion is occurring in GHCS [8]. While there is a growing "globalization" literature for industry in general, which documents cases, methods, best practices, and an emerging body of theory, there is little work on the differentiating aspects of GHCS [9].

Health care services can include an array of goods and services including diagnostics, pharmaceuticals, medical supplies and management services for health care organizations. For the purposes of this paper we focus upon the delivery of treatment to patients: the therapeutic activities of provider to patient and its organizational setting, in this case the King Hussein Cancer Center (KHCC). Non-health care product and service sectors have recognized the specialized demands on the expatriate or international manager to effectively operate in a foreign setting. There is a resulting human resource development literature regarding the capabilities and training for global assignments, and a managerial effectiveness and leadership research literature informing those training and preparation activities [2]. While there are some useful cases and emerging models [10], there is a paucity of research and resulting literature on leadership in global or international health care settings and less regarding the leadership capabilities required to increase the likelihood of success in the GHCS context. Filerman [11] has called for the application of "transformational leadership" to achieve success in GHCS, yet there is very little existing health care literature describing the capabilities, mechanisms and contexts which support this admonition.

The article describes changes which occurred at KHCC between 2002 and 2005. It provides insight into the unique leadership challenges of GHCS and specifically offers observations on the transformational leadership model. During this three year period KHCC was able to: 1) grow in numbers and types of services, 2) achieve certification by an international accreditation body, and 3) reach fiscal balance and accountability. The analysis of the case study suggests that the behaviors of transformational leadership were strongly associated with these changes. It was necessary to draw on literatures outside transformational leadership to adequately describe the "transnational culture" and "political competencies" observed at KHCC. The analysis and discussion sections suggest an expansion of the transformational leadership framework in GHCS suggesting new avenues for research in global health care leadership.

## Methods

There is a long tradition of case research in medicine and business which seeks to describe, understand and explain phenomena. The "exploratory" case research method used in this study finds its rigor through corroboration by multiple sources (e.g. interviews, documents, direct observation), richness of insight, and provision of multiple explanations for the same phenomena [12]. KHCC has a useful set of written documentation and evaluation reports [13-16] developed at the onset of the institutional change initiatives beginning in 2002. This allowed the research team to review written accounts, look for confirmation in interviews and, in some instances, to guide direct observation at the facility.

The researchers and a research assistant travelled to Jordan for staff interviews on June 5 – 12, 2005. The site visit was preceded by telephone interviews with four staff members and multiple telephone discussions with the Director General, Dr. Samir Khleif. Approximately 15 interviews were scheduled before the team arrived in Amman and 13 were added as the team followed the thread of the inquiry. Given the exploratory nature of the case study, the researchers did not have an *a priori *theory to test, but used open interviewing technique to build a data set of anecdotes, historical recollections and personal observations of interviewees. The researchers asked Dr. Samir Khleif to review the case study portion of the manuscript for accuracy. Dr. Khleif made no comments on the analysis and there was no influence on the researchers regarding their interpretation of interviews, events or reports that were used as source materials. Interviews were digitally recorded with the consent of each interviewee, and transcribed using software (Dragon) and human interpretation.

## Results

### Case study

This brief narrative of the critical events at KHCC between November 2002 and March 2006 provides the facts on which the observations in the discussion section are made. On February 16, 1999 King Hussein of Jordan died of cancer after a long series of treatments in U.S. cancer centers. High level relationships between the royal court of Jordan and the U.S. government led to proposals for cancer related projects including creation of a U.S.-style cancer treatment center [17]. This model for cancer treatment, based on comprehensive, evidence-based, patient-centered care, was to replace the poor quality and low levels of service that were available in the country. Baseline assessments of the cancer treatment in Jordan showed major problems with quality of care, a lack of full-time leadership and serious safety issues.

During negotiations between the Jordanian royal court and the U.S. Health and Human Services National Cancer Institute, Samir Khleif, MD, was selected to lead the transformation. With a personal history in the region and affiliation at the National Cancer Institute (NCI), Dr. Khleif was a highly desirable candidate for assignment to this project. Dr. Khleif negotiated two critical pre-requisites as contingencies for his acceptance of the project leadership: 1) a bank account with a settled-upon amount for investments into the facility and staffing at KHCC with no restrictions on Dr. Khleif's choices and timing, and 2) autonomy in all management issues, specifically the firing and hiring of staff and purchasing of equipment and materials. On November 16, 2002 Dr. Khleif took the position Chief Executive Officer and Director General (DG) of Jordan's major cancer hospital with his negotiated funds and management autonomy. The case study proceeds as a series of phases or stages. These stages were both planned and emergent. They were partially anticipated, according to Dr. Khleif, and emerged *in situ *as the leadership team collectively envisioned, planned and executed the changes.

### Inception phase: do no harm – "the war room"

The first task was to establish what was termed a "safety agenda." The external evaluations of the Jordan cancer hospital had revealed a number of dangerous conditions in the hospital including no rails on children's beds, no emergency response team in the hospital, dangerous and inappropriate mixture of chemotherapy, and problems with infection control. The safety issues provided legitimacy for a "shock therapy" approach to the transformation that followed. During this inception period, the option of accepting no new patients was strongly considered due to the level of safety concerns. New patients were accepted given the high unmet need of newly-diagnosed patients and the judgment that the most immediate and serious safety gaps could be closed in short order.

Selection and recruitment of key staff was a critical initial step. The DG's pre-existing reputation both in the region and at NCI, coupled with his negotiated autonomy and resources, allowed him to recruit staff with clinical and technical excellence and knowledge of what can be called a "U.S. Cancer Center Model." A team of technical experts (U.S. and European-trained Arab region professionals with credentials in all aspects of cancer care and hospital management) was recruited from within Jordan and from abroad to implement the changes. A "war room" was created in which daily meetings allowed flexible decision-making. Strategic intent and evolving objectives (e.g. aspirational goals v. deadline driven objectives) guided the decision-making process. Decisions were made on strong technical guidance (the vision of a U.S. cancer center) and appreciation of opportunities as they arose.

Other initial steps included creation of financial accountability and controls and incentives for the senior staff. Strategies were devised to train existing staff to improve processes and implement new programs. Dr. Khleif and other interviewees reported intentional commitment and references to quality improvement processes outlined in the Joint Commission International accreditation [18]. In-service trainings were used to set up new systems that provided adequate levels of re-enforcement to integrate new operations and services while care delivery continued. Much of this training was provided during short term consultancies from international experts (medical, nursing, pharmacies, laboratory). A few key members of the KHCC staff were sent abroad for short courses in critical areas.

Symbolic (and critical to the inception phase) was changing the institution's name from the Al-Amal Center to KHCC.  (pronunciation: al-ahml) means "hope" in Arabic. The perception of the center caused local residents and patients to refer to it as  (pronunciation: al-haml) which means "bums." The pejorative slang described the center in one word as poor quality and operated by incompetents. Using the name of the honored and recently deceased King to re-create and renew the center was done with the express intent of both de-stigmatizing cancer treatment and suggesting a transformation from poor to world-class quality in cancer treatment.

#### Rapid scale-up of quality services

During the second phase the number of patients, patient services, and programs increased rapidly (exponentially) with a proportionate increase in staff. The transition included significant turnover among the original staff. The Al-Amal staff who were retained were those who expressed a desire to support significant change and who demonstrated a capacity for improvement. Interviewees reported that as the demands for change increased, job satisfaction concomitantly increased, due in part to greater training and higher expectations. They also reported significantly greater demands on their time and a sense of shared commitment to the achievement of organizational goals.

A "war room" approach to managing change was utilized where the senior staff met daily after completing their usual duties to discuss and create the emerging plan for transforming the center. The war room evolved over time into operating committees based on structures that exist in hospitals and cancer centers in the U.S. and Europe. The rapid scale-up also was marked by development of guidelines, protocols, and standard operating procedures. Major emphasis was given to the development and coordination of support services (lab, pharmacy, infection control) that typically are lacking in hospitals in developing countries. Development of a multi-modal approach to patient care using clinical teams ended the "one man show" approach to patient care that had previously dominated cancer care at Al-Amal.

Modern hospital management techniques were implemented, including a shift from inpatient to outpatient; introduction of process management; data systems to manage length of stay (inpatient and outpatient bed use, waiting times) and redesign of bureaucratic requirements to enhance patient satisfaction and efficiency (responses to patient complaints or improvement recommendations reduced from 18 steps to 3, elimination of multiple stamps by issuing insurance card). New services were added that ensured comprehensive and quality cancer services at KHCC, including palliative care.

During this period, department plans and budgets were used as a loose guide to a negotiated process for implementation of changes. This flexible approach allowed the management of the center to take advantage of opportunities as they arose (e.g. the availability of a unique staff candidate, or accommodation to bureaucratic needs of the Ministry of Health). Systematic training replaced the ad hoc approach to address both the needs of new programs and the safety agenda. Orientation for new staff and preceptor training was implemented. Continuous Medical and Nursing education was begun.

#### Maturation phase

The maturation phase of development can be marked by the beginning of the process toward which KHCC sought international accreditation. The Joint Commission International (JCI) Accreditation [18], an international recognized body that certifies quality of care in patient care institutions including cancer centers, would confer their accreditation if KHCC met its international standards. In the summer of 2005, KHCC conducted a "ghost evaluation" of itself in preparation for the visit of the evaluation team.

An overt commitment to JCI processes and principles was made at the beginning of Dr. Khleif's term as DG. In presentations to staff he identified a four-fold rationale for following JCI. 1) The process can be learned, measured and applied to the specific challenges facing KHCC. 2) JCI accreditation itself, while a desirable goal, is a by-product of the primary aim to have the institution commit to a continuous quality improvement process. 3) JCI is sustainable and valuable for the long-term benefit of the institution whether accreditation itself is achieved or not. 4) JCI is a proven international process and standard.

The maturation phase was also marked by internal recognition of an impending challenge to quality due to the rapidly increasing patient load at KHCC. Successful cancer therapy leads to longer survival of patients and the accumulation of patients who need on-going evaluation and therapy. Even without increases in new patients, a successful cancer center will increase its patient visits and load because of the way that cancer has been transformed into a chronic disease for many. The limitations of the physical plant at KHCC created a limit to the number of patients that could safely be treated. By June 2005, KHCC had made the decision to limit new patients until the physical infrastructure could be increased with the building of a new structure. A balance between expenses and receipts had been reached based on the levels of patients being treated; indeed, financial surpluses were posted in 2004 and 2005 (see Tables 1, 2, 3).

**Table 1:** Outpatient visits, new cases and employees at King Hussein Cancer Center, 2002 – 2005.

	**2002**	**2003**	**2004**	**2005**
**All Outpatient Visits (Adults, Ped., Mix, BMT)**	10,870	25,539	45,523	60,433
**New Cases**	1,040	2,316	2,896	2,772
**Employees**	570	772	942	1,129

**Table 2:** Revenues and surpluses at King Hussein Cancer Center, 2003 – 2005.

	**2003**	**2004**	**2005**
Revenues*	20,274,331	33,266,489	35,266,489
Surplus*	2,698,053	7,644,744	7,998,885

*Shown as Jordanian Dinars (JD)

**Table 3:** Performance indicators at King Hussein Cancer Center before and after transformation.

**Performance Indicators**	**2002**	**Jan-June, 2004**	**July-Dec, 2004**
Average length of stay (in days)	12.1	6.8	6.0
Average length of stay without the BMT (in days)	11.9	5.5	4.8
Attending physicians progress notes	< 10%	71%	82%
Wasted x-ray films	7.8 %	4 %	2.6 %
Post-operation order documentation	< 60%	72%	74%
Cancelled procedures in operation room	28%	18.6%	16.1%
Scheduled Visits	10%	80%	90%

KHCC began to reach out to the broader medical community in Jordan and the Ministry of Health. Having established itself as a center with international ties and improving quality, other facilities and bodies in the country began to approach KHCC for assistance and advice. KHCC provided technical assistance and recommendations regarding long-term training, development of professional standards, development of civil society (volunteerism, stigma reduction), support of national policy reforms to support national development, and improved integration of KHCC into the national referral network. The next stage of KHCC's development (participation in international collaborative clinical cancer research) was inaugurated with the establishment of an Institutional Review Board, to ensure the ethical treatment of the human subject, a pre-requisite for participation in international research.

The final challenge in the maturation phase was the identification of a leadership succession process. In late June 2005, the Board of KHCC called the senior leadership team together to announce that the DG would be returning to a post at the National Cancer Institute in February 2006. They announced that an international search would begin to find his successor.

Subsequent to the data collection in June 2005, KHCC was evaluated by the Joint Commission International (JCI) site team in February 2006. JCI awarded KHCC its accreditation at the conclusion of its site visit. Dr. Khleif's successor was announced who took responsibility as the Director General effective March 1, 2006. Samir Khleif returned to the National Cancer Institute having completed his KHCC assignment.

### Analysis: transformational leadership, transnational culture and political competence explain rapid changes at KHCC

Reviewing the interview and other data led the investigators to three concepts from the available literature which had the power to explain the speed and depth of change observed: transformational leadership, transnational culture and political competence. Transformational leadership has included both "culture" (as values, honesty, approachability) and "political" (sensitivity and skills) in its formulation [19]. However, the observations are primarily within-organization focused: the norms, values and preferred behaviors of an organizational culture; the internal negotiations, influence and relationships influencing access to scarce resources in the political dimension and "setting boundaries" with the external environment. KHCC observations lead one out of the institution and into a cultural network that is regional, international and professional; political dimensions that are societal and cross national borders, not to constrain but to expand the boundary of the institution. Dickson et al [20] have asserted that while new work on the relationships between society and organizational culture are emerging, most work has focused on "the measurement and description of relationships, without specifying the mechanism by which the influence is enacted." Those mechanisms, as reported by KHCC interviewees, were in the behaviors and capabilities of the leadership.

Transformational leadership has been researched across a variety of international settings [21] including health care. Pawar [22] has asserted the context or circumstances inciting organizational change are not fully understood in its relationship to transformational leadership where "research suggests different positions on whether transformational leaders focus on attaining change mainly in followers or in institutions or both." KHCC can provide directional insights to the relationship among the leader, the followers and the results; the globalizing health care context and a change goal of achieving improved, international standards of care in a developing country.

#### Transformational Leadership observed at KHCC

Leadership emerged as a predominant theme when the staff of KHCC were asked how the changes emerged, why they succeeded and to what degree. Repeated and lengthy descriptions of leadership, frequently naming Dr. Khleif or one of his direct associates, were described as the causes of the changes. The leadership qualities reported included the ability to draw out of themselves and their followers, significant sacrifices that went beyond their own self-interest. There was a sense of purpose or vision-driven efforts to attend to the needs of patients and mid- and lower-level hospital staff (training and soliciting their suggestions for improvement) in an effort to rapidly raise the standard of care at KHCC. These experiences of followers and the persuasion abilities of leaders are hallmarks of "charismatic/transformational" leadership [23,24]. Proponents of this leadership theory have conducted cross-cultural studies suggesting that transformational leadership attributes are universal [25-27]. They qualify "universality" arguing that the attributes are mediated by the culture-specific expectations of the followers and the description of these model-derived behaviors can vary widely when subjects from different cultures describe the effective and ineffective behaviors of leaders [28,29]. As Hartog [26] described, "although concepts such as 'transactional leadership' and 'transformational leadership' may be universally valid, specific behaviors representing these styles may vary profoundly." Dickson [30] differentiates between "simple universals" which do not vary from culture to culture and a "variform" universal where culture-specific subtleties to the universal are observed. It was outside the scope of this research to specifically differentiate these contrasting forms. However, insight into the KHCC variforms may be derived from the discussions of cultural sensitivity and political competence.

Leadership, as described by the interviewees, was not characterized in culture-specific terms. There was no report that the DG or his leadership team were "Arab" or "Jordanian" or "American." There were characteristics of the new leadership team that interviewees described as contrasting sharply with the previous administration of the Al-Amal hospital. Given the research team was Western, likely identified as "American", it is possible a "demand characteristic" was created where interviewees would be reluctant to share culture-based criticisms or observations with the researchers. It is also possible the researchers were unable to see or misinterpreted certain culturally embedded information.

KHCC leadership was reported as being both "goal oriented" (towards the rapid achievement of much higher standards of care, and improvements in organizational functional departments and practices that support direct patient contact) and "participative" (followers were involved, in some cases reported as being "required" to participate in identifying new practices, new improvement process, etc). Bass [21] has noted that transformational leadership can be "democratic" or "autocratic". KHCC interviewees reported the leadership was involving, participative, yet with an unwavering resolve toward higher standards of care.

KHCC interviewees reported aspects of the leadership they observed or participated in which fell into four components of the transformational leadership that have been identified in previous research [23]: "inspirational motivation", "idealized influence", "individualized consideration" and "intellectual stimulation."

Following Avolio [31], leaders create "inspirational motivation" when they articulate a future state of the organization that is appealing and inspiring which seeks new, higher goals or standards. They express optimism that the goals can be attained, which serves to give a context of "meaning" when members of the organization are asked to make sacrifices and/or work through difficulties. Many members of the senior leadership team who had received additional medical training outside the Middle East reported they came to KHCC for less pay than available at other postings and it was uncertain whether the KHCC experience would enhance their chances for future assignments at greater levels of pay, increased responsibilities or at more prestigious institutions. It was frequently cited that moving toward higher standards of care required significant personal and group sacrifice. Daily meetings going late into the evenings for making decisions, solving problems, and building the emerging vision of the new organization were reported as requiring significant personal sacrifice but also being motivational. Western consulting and hospital-based evaluation teams provided strong evidence that patient safety was in jeopardy. These studies were accepted and created staff commitment to new, higher goals rather than creating cynicism or a fatalistic response. The senior leadership team assembled by the DG were able to frame these gaps as inspirational and motivational, although closing them would requiring significant levels of personal and group sacrifice at all levels of the organization.

"Idealized influence" is a process in which followers identify with the leader and strive to emulate or admire him/her as an ideal. The leader demonstrates conviction, takes stands, and makes appeals of an emotional nature. Many interviewees reported a personal admiration for the DG and other members of the leadership team. In many instances collegial relationships had begun at other medical institutions or in earlier training. Some reported that the appeals to join or remain on the KHCC team were emotional and "irresistible". Beginning with the DG and in the behavior of other members of the leadership team, they reported a very high level of commitment to professionalism, a willingness to take stands about patient care and against incompetence. An event with high salience for many interviewees was a pivotal "sacking" that occurred in 2002 where a direct report to the new DG was let go. It was believed that highly placed friends or relatives would intervene on this individual's behalf and he would be re-instated. The fact that the DG's decision stood was seen as a demonstration that he had conviction, and could take a public stand and prevail. These and other similar observations also suggested a separable capability, "political competence" which is discussed later in the analysis.

Interviewees frequently referred to the perceived "respect", deference shown to them as professionals, reported most frequently as behaviors of the DG. Respect was also demonstrated through the involvement of the staff in the emerging vision for the new KHCC. Rather than imposing a detailed plan based on experiences outside Jordan, the specific goals were expressed as aspirations and staff at the top management levels had direct and significant involvement. Training, based on individual development needs for staff at all levels, was encouraged and provided. Team building, specifically among the top leadership team and at the unit or department level, served as a vehicle for gathering ideas, consolidating commitment to plans and reinforcing mutual respect. These observations are consistent with two additional components of a transformational leadership model referred to as "individualized consideration" and "intellectual stimulation." This is leadership behavior which attends to individual needs, specifically incorporating and engaging concerns, challenging assumptions, and soliciting the ideas of others. While the DG had a broad strategic intent which he expressed to his senior staff and their direct reports, he reported a conscious effort to be vague in the specifics to allow others to have significant involvement in the emerging vision and plans for change and due to his own belief that what would be successful at KHCC could not be known prescriptively, *a priori*.

#### Cultural sensitivity as a health care leadership capability

"Cultural sensitivity" or competence was frequently cited as an explanation for the changes at KHCC. In the Western health care literature, "cultural competence" [31] has been identified as valuing and understanding other cultures, and acquiring a base understanding of the norms, preferences and biases that can influence effective patient/provider interaction. The majority of this literature describes Western settings where patients from other nationalities and social cultures are seeking care, with prescriptive techniques based on case examples for the individual care provider to become more culturally sensitized and as a result more effective [32,33]. There is a strong focus on the individual provider v. the institution or the leader/manager. In contrast, the international management literature emphasizes cultural awareness and sensitivity as a leadership capability or competence associated with leading the overall success of the enterprise. At KHCC, cultural sensitivity was reported in relation to the development of Western medical clinical professional norms, the patient/provider relationship, the leader/follower relationships and the extra-mural relationships between KHCC and within-country and across border organizations. These suggest awareness and competence with a range of issues that is broader than the current description of cultural sensitivity in the transformational leadership literature.

Moore [34] has suggested that "culture" in transnational organizations is a complex, shifting concept and that transnational business organizations reflect neither "national" nor "organizational" cultures, but a blend. Observations at KHCC lead to a similar conclusion where KHCC was a complex blend of local and global; where the organization incorporated into itself aspects of the organizations and oncology leaders with which it transacted. The technology itself which supplied the methods of communication and its content influenced the culture of KHCC. This conception of organizational culture had more utility for understanding the changes at KHCC and has been referred to as a "third cultures" perspective in the business culture literature [35].

Three cultures were identifiable: local societal (Jordanian/Gulf region), global international (American/Western) and professional (medical clinical/scientific). The separable aspects of each culture were observed at various physical locations or in specific tasks. Examples observed were "local" culture in the patient/nurse or patient/family/nurse encounter or between the KHCC leaders and the Ministry of Health; "Western" culture in the information systems and the video-conference room where KHCC staff interacted in real time with staff at St. Jude's Hospital in Memphis, TN or the National Cancer Institute in Bethesda, MD; and "professional" culture in language choice for record keeping (English) and in interpreting diagnostics, selecting treatments and international contacts with other leading oncology practitioners and institutions. This cultural blending view follows Morgan [36] and Doz et al [37] who invoke the terms "transnational" or "metanational" to describe multi-national organizational culture.

Four clusters of observation describe in more specific detail the transnational culture at KHCC. They provide additional support for a broader re-conceptualization of cultural sensitivity and capability; suggesting the characteristics, capabilities and contexts for leadership selection and training. The four KHCC circumstances observed were staff recruitment, end-of-life care, language, and communications technology.

#### Cultural competence as a criterion for recruitment

Recruiting senior staff with a sensitivity to and tacit knowledge of the local culture has been identified as an important success factor for expatriate managers [38]. Of the 23 members of the top management team, all were Gulf region nationals (Jordanian, Syrian, Saudis) and all have professional training, degrees and/or certificates from Middle East institutions. Nineteen of the 23 had additional advanced degrees, board certifications, training or certificates from Western institutions (e.g. U.S. or U.K. schools of medicine or other disciplines; in some individual cases more than one degree or certificate). Advanced training also suggested that the leadership team was highly qualified and competent as clinical scientists familiar with the new practices and therapeutics in an operating clinical oncology setting. They had personally succeeded in high performance and high technical medical environments within and outside the region (see Table 4).

**Table 4:** Qualifications of senior staff, King Hussein Cancer Center

# Senior KHCC Staff (%)	**23 (100%)**
# Senior Staff with Middle Eastern Advanced Medical Professional Training (%)	**19 (83%)**
# Senior Staff with Board Certification or Certificates from Middle Eastern Institutions (%)	**8 (35%)**
# Senior Staff with US or EU Professional Degree (%)	**10 (44%)**
# Senior Staff with U.S. or EU Board Certification or Certificates (%)	**16 (70%)**

Recruiting staff with this profile ensured they functioned as part of a global medical society, understood the high standards of clinical expertise required by that informal society, as well as having significant experience with the norms and customs associated with Western technology and communication. Their backgrounds as nationals from the region also ensured they had awareness of local and regional customs as well as religious norms. Multi-national corporations have used a similar practice of hiring local nationals with expatriate experiences or education in the West. Vertovec [39] and Moore [34] suggest organizations which have followed this employment pattern create a cultural "trialectic" of local, Western and a third culture, the global. Following Moore we speculate the global, in this health care case, can be described as a "global medical society." It suggests the senior staff recruited to leadership positions at KHCC had significant training, experience, professional relationships and interests that transcend Jordan, the U.S or any national border. They are perhaps more aptly considered members of an informal "society." To understand the role of culture and the motivations of the leaders, it was more useful to consider the KHCC leaders in a global oncology society who both influenced that network of oncologists and were influenced by it.

#### Managing End-of-Life Care: an exercise in cultural competency

End-of-life care is a critical component of quality cancer care (as a component of palliative care) and is highly culturally sensitive. Quality end-of-life care did not exist at Al-Amal. Translation of the guidelines and standards of palliative care into an Islamic context was a necessary step. The existing system in Jordan at the time of the transformation sought to prolong life at any cost (even when no effective options existed) and paid secondary attention to quality of life of the patient (e.g. pain control). KHCC staff with training in palliative care were able to introduce progressive changes in practice by understanding the culture of Jordan and Arab Islam enough to mobilize and create cultural support. One interviewee provided the following example.

"Many of our patients said it was against Islam to let people die. We explained that Islam teaches us that we should seek and apply knowledge to help humanity. Our prophet taught us that we should go 'even to China' for knowledge. The meaning of this is that we should strive our utmost to learn how to help people. Our patients understood and accepted this reasoning and supported the palliative care we offered."

This sort of reasoning, as well as knowledge of cultural context, successfully led to cooperation avoiding cultural misunderstandings or confrontations.

#### Language as culture and management tool

Cultural competence by KHCC leaders can also be illustrated in the use of language (Arabic and English). Patient charts were in English. In some instances there were additional handwritten notes reflecting or supporting patient interaction in Arabic. Arabic was used as the primary language for patient, family, and local physician interactions; training and engagement with staff in the clinical setting; and intra-region interactions. War room discussions by the leadership team were conducted in English as well as international clinical interactions. It was reported that some euphemisms or "short hand" expletives and phrases used in the war room were Arabic; their usage may have increased in times of stress or disagreement although interviewees were not definitive in their recollections. KHCC staff used English or Arabic to fit the needs of the clinical situation and this bilingual fluency was critical to the unique trialectic of local, Western and clinical scientific cultures that supported rapid change.

#### Managing communications technology as cultural competence

The ability of KHCC management to deploy information technology was reported and interpreted as a cultural competency. KHCC, before the transformation, had a poorly developed "information culture." More widely distributed and greater information technology capability was a priority during the period of rapid growth. Increased email and internet access, video conferencing, TELESYNERGY Global Medical Consultation Workstations, video conferencing, access to the National Institute of Health (NIH) Library, "tele-pathology" and access to NIH video casts were all made possible. There was a three-fold increase in the total number of personal computers (PCs) and particularly fast-processor PCs along with printers. Electronic billing and scheduling systems were also deployed. The increased use reinforced the transnational culture, especially as it introduced Western/European modalities and assumptions through interfaces, acronyms and assumptions about data handling and use. The implementation of this technology was for the purpose of enhanced patient care. Viewed as outputs, these exchanges also brought KHCC into a community of advanced Western cancer centers. One by-product of joining the global community of care centers was KHCC became more attractive to regional medical students as a location for residency. In 2004, KHCC had 160 applications for its internal medicine residency program. By attracting more local medical students, it supported KHCC's aspiration to be self-sustaining over time, as regards medical manpower, and to have a broader impact on the region by increasing the number of highly trained physicians.

#### Political competence necessary to success at KHCC

Ferris [40,41] has argued that political competency is required in global settings due to the uncertainty and variety that expatriate managers experience in global assignments. Failure of overseas expatriate managers has been associated with a lack of political awareness and skill [42,43]. The "health reform" literature [9], while focused on broader health system reforms, emphasizes the importance of political understanding and strategy in order to succeed; yet with no indication of the specific knowledge, skills and capabilities required.

Ferris has identified four knowledge, skill and ability clusters associated with political competence that were found useful to understand and interpret the KHCC observations: self and social awareness, interpersonal influence and control, genuineness and sincerity, and social and political capital inside/outside the organization.

"Self and social awareness" suggests an awareness of the impact of one's behavior on others and in turn accurately interpreting the behavior of others in a social situation. While no one KHCC interview identified this component, the research team observed this type of reflexive knowledge among several of the interviewees and the DG in particular. While a weak finding, the data supports "self and social awareness" within the construct of political competence observed at KHCC.

"Interpersonal influence and control" is the ability to foster a sense of trust and confidence in others. Others confer these upon the leader which creates a willingness or explanation for their willingness to follow. This may be of greater utility in an expatriate setting where there is more uncertainty. The KHCC data suggest the DG demonstrated this competence in his ability to recruit a powerful cadre of global elite physicians. The local community and staff were aware the DG had been recruited with direct involvement of the Jordanian Royal Court. This conferred upon him and his delegates access, power and influence in the wider community. His negotiation for upfront guarantees of control and resources also suggested he had the necessary political "capital" to be successful in negotiating with authorities and entities which had direct or indirect influence over KHCC.

"Genuineness and sincerity" in Ferris' usage is the ability to effectively use the social norms of the expatriate culture to project a sense of authenticity in interaction. We observed the DG in particular and other members of the senior management team as demonstrating an authentic personal commitment to the changes and resulting incremental achievements at KHCC. While there were reports of disagreements and personal preferences for some individuals regarding his/her leadership style, there was a uniformity of recognition that the degree of personal sacrifice and hard work demonstrated this dimension of genuine commitment and sincerity.

"Social and political capital inside/outside the organization" is the ability of the expatriate to harness useful external relationships and meld them with internal resources toward the organization objectives. It is unequivocal in our findings that relationships held by the senior management team with overseas and domestic health care organizations were of vital importance to the success of KHCC. These active relationships with the global network of cancer centers and cancer advisory groups (i.e. National Cancer Institute) made it possible to effectively use technology which facilitates the transfer of knowledge globally. The technology facilitates the communication, but leaders within the health care institution required political currency and competence to fully access the global knowledge.

Political competence in the health care setting also has direct operational influence over the financial health of the institution. Incoming cash flows from government controlled reimbursements bears out this point.

In 2002 KHCC found itself with accounts receivables:

Palestinian Authority 27.2%

Jordanian Government 58.2%

Libyan Government 5.6%

Algerian Government 2.6%

Other (Firms & private patients) 6.4%

This led the Ernst & Young report [13] to offer that "due to political situations in the Middle East ... a delay in collecting the amounts due ... will probably take place." Negotiating the collections of these outstanding receivables in a timely manner requires political acumen and competence. Interviews underscored the importance of this competence to explain the rapidity and sustainability of change. It is noteworthy that among the reforms at KHCC were "Western-style" credit controls which decreased doubtful receivable accounts to 1.2%.

## Discussion

The research team found greatest utility to explain the changes at KHCC in a Western-derived model, transformational leadership. Within the transformational leadership literature, there are alternative formulations [44,45] that vary somewhat from the Avolio and Bass model. It was beyond the scope of this research to determine which variations within the transformational framework had stronger empirical support. The Avolio [23] four "I" factors of "inspirational motivation", "idealized influence", "individualized consideration" and "intellectual stimulation" were congruent with the interview data. None of the interviewees, including the DG, reported any training in leadership theory or a model of organizational change they were following. The experience reported by interviewees as well as other sources made available to the research team suggest the leadership made changing the attitudes, skills and motivation of staff at KHCC their means to the institutional changes they sought. This finding would suggest that in the KHCC case, effective transformational leadership focuses on changes among the followers as the means to institutional change [23]. It is less clear from the KHCC case whether accreditation v. adhering to JCI continuous improvement practices was the primary institutional change goal. Both were achieved and therefore confound an analysis as to whether staff accepted leadership's espoused prioritization that adherence to practices was superior to JCI accreditation. One can speculate that staff commitment to JCI processes could decline if accreditation had failed.

There are other frameworks to understand leadership that might be considered to understand the changes which occurred at KHCC. Leadership models derived from within the Arab culture [46,47] can serve as counterpoints to the transformational model. A review of the literature identified management theory derived from the Arab region [48].

Ali suggests that the case for Arab-specific management theory grows out of the unique religious and cultural history of the region. He suggests that Arab management theory is in its infancy, and that political, economic and social forces influence it both toward and away from adaptations of Western management theories. For example, he argues that Arab management has been traditionally tribally oriented and "manager and organizations exist to further the interests of a collective group (individual, family and layers of tribal network)." This view has been identified as the "sheikocracy" leadership style [46] with its high degree of paternalism, bureaucracy and dependence upon personal and tribal connections. The KHCC management team reported self-conscious steps to specifically differentiate itself from this culturally-specific style of leadership. The KHCC leaders expressed a concern that staff who believed they or others were in positions due to their tribal connections, would be unable or unwilling to make the significant changes and sacrifices required at KHCC. Tribal connections are not performance characteristics and therefore undermined a move toward a more performance-based and measurement oriented approach to management.

Another alternative Arab leadership form is described by Khadra [47]. In his "prophetic-caliphal" model, a prophet emerges who has the ability to accomplish a great goal. Khadra suggests that followers will make profound personal sacrifices in the belief the leader is a great man who has appeared to perform a "miracle" which is linked to their own personal ideals. It is possible and plausible that reported behaviors of the leaders at KHCC, which are interpreted as universal manifestations of transformational leadership can be interpreted as Khadra's "prophet". Nothing in the KHCC data suggests the leaders couched their decisions, plans or activities in religious terms or intent; identified themselves or were identified as "prophetic", but these cultural archetypes may have been aroused.

While the KHCC case appears to confirm the universality of transformational leadership, some have argued there are no global leadership theories [3]. They argue leadership is too socially/culturally embedded and explanations taken from outside the local social culture may be reductionist or translations that obscure important variables. Our findings support application of the transformational leadership model in a non-Western setting. Culture was observed as broader than the intersection of national social cultures. It can be argued, based on our findings, that global leadership theories are indeed inadequate without a broader accounting for the impact of culture imbedded in Western technologies facilitating globalization, and with the professions, like medicine, whose knowledge is crossing borders bearing its own cultural ethos carried by its global social networks of sub-specialty clinicians.

The KHCC analysis suggests GHCS may have unique properties that differentiate it from private sector health care globalization. The KHCC case demonstrated three aspects of global health care trade which are associated with health care globalization: "cross-border supply" (medical diagnostics, interpretation and guidance transcending national borders, e.g."telemedicine"), "consumption abroad" (patients traveling across national boundaries to receive care), and "movement of health professionals" (health professionals who voluntarily seek overseas employment or are contracted to overseas work) [7]. We suggest that the fourth element in private sector global health care trade, "commercial presence" (foreign direct investment in health products and services), can be augmented to include government and non-governmental economic and human resource exchanges. Broadening this fourth element to include government involvement, such as in the KHCC case, may generally increase the political dimension in GHCS and therefore requires a wider spectrum of leadership capabilities which we have described as "political competence."

The analysis of this case study focuses on agency (leadership, management, the actions of individuals) and has not addressed issues of the enabling environment. TTThe enabling environment for the KHCC case includes factors related to the health service delivery market, the institutional context in which KHCC exits, and a broader geopolitical context. The existence of a fluid and resource-rich regional health care industry explains how KHCC could draw upon trained manpower, critical medical supply markets, and a local/patient base. At the institutional level, the changes at KHCC must be understood within the context of longstanding relations with the royal court, a Ministry of Health with its own policy environment, and professional network with its own power base. The KHCC is in the public sector and has operational interactions with private sector firms. Finally the global geopolitical context of KHCC cannot be minimized. A beloved King who died of cancer, his widowed Queen with strong U.S. ties, and a supportive U.S. Health and Human Services senior leadership with aspirations to play a stronger role in global health were some of the critical factors that led to the developments at KHCC. A full discussion on the role of the enabling environment around KHCC would require an analysis of much greater length. This paper has a more narrow focus: leadership activity associated with the changes that led to improved clinical standards of care.

## Conclusion

The case study narrates a series of organizational changes that are highly contextual to the specifics of the KHCC and Jordan. The case study provides a point of comparison with other cases of transformational leadership and supports the broad utility of that theory in an international health care setting. Our conclusion, based on all the data sources, is KHCC leadership focused on changing the knowledge, skills and attitudes of the staff to achieve their aim of institutional change. Revisiting Pawar's [22] question on the aim and context of transformational leadership: institutional change v. change in followers; the case would suggest the successful transformational leaders at KHCC focused on followers as the means to achieve institutional goals. At KHCC, adoption of JCI processes were the primary stated aim and accreditation espoused as a highly valued, but secondary goal. The KHCC observations of culture and political competence required sources outside the existing transformational leadership literature. Culture, in the GHCS context, extends beyond institutional boundaries to include the role of professional culture and cultural content that is carried within the communication technologies that make globalization possible. Probably due to the role of governments in domestic health care, and in the exchanges of resources of personnel and resources in the KHCC case, political competence has particular utility as a leadership characteristic. Since many GHCS initiatives include government involvement, it suggests further research should focus upon political capabilities that are broader than boundary setting with the external environment. Political capabilities can have specific impact on the financial health of the institution and ultimately on the achievement of change goals.

Other leadership theories derived from the Middle East were useful counterpoints and may plausibly explain some of the observations. This study, which focuses on agency, has only touched on the enabling environment which must also be appreciated to fully understand KHCC's achievements. Enthusiasm for the "best practices" emerging from this successful GHCS must be tempered with an appreciation of the unique set of features in the enabling environment of this case study. Nonetheless, the successful development of a cancer center meeting international standards in a developing country should give encouragement to many working to address the pressing health needs in developing countries.

It must also be noted that changes in staff motivation and achieving institutional goals over a three-year period, while encouraging, may not persist. Leading an institution to achieve near-term goals can provide useful insights, but sustaining high levels of clinical excellence over an extended period of time would provide significant additional strength and support to the KHCC observations. An important future marker will be the required follow up JCI evaluation of KHCC in 2009.

Finally, political competence and resulting influence is notoriously ephemeral. A transformational leader with significant political competence may find his reach and grasp lessen or even disappear as the political context evolves. Political competencies should be identified, communicated and taught; leaders selected and trained to possess them. But those capabilities will always be context-specific. The observed changes at KHCC, due in part to political competence, may not have been possible if there were a different political context in 2002 – 2005, irrespective of the leaders' skills; and may not be replicable beyond those years due to forces beyond the control of any individual leader of a health care institution.

## Competing interests

The author(s) declare that they have no competing interests.

## Authors' contributions

Each author contributed to the concept and design of this study, participated in the site visit, analyzed the findings and participated in manuscript development.
